# Analysis of pediatric assent information in early-phase cancer clinical trials through a children’s research advisory group

**DOI:** 10.3389/fpsyg.2025.1655835

**Published:** 2025-10-03

**Authors:** Ana Mínguez-Garrido, Valeria Moriconi, Beatríz Vergara-Muñoz, Susana Buendía-López, Maitane Andión, Blanca Herrero, Mirian Luengo, Alba Rubio-San-Simón

**Affiliations:** ^1^KIDS Madrid, Unidad de Ensayos Clínicos, Fundación Investigación Biomédica Hospital Infantil Universitario Niño Jesús, Madrid, Spain; ^2^Universidad Europea de Madrid, Villaviciosa de Odón, Spain; ^3^Aladina Foundation, Madrid, Spain; ^4^Clinical Trials Unit, Paediatric Haematology-Oncology Department, Hospital Infantil Universitario Niño Jesús, Madrid, Spain; ^5^Paediatric Haematology-Oncology Department, Hospital Infantil Universitario Niño Jesús, Madrid, Spain

**Keywords:** Pediatric assent documents, clinical trial communication, young persons’ advisory groups (YPAGs), patient-centered design, youth engagement in research

## Abstract

**Introduction:**

This study aims to identify potential barriers to children’s proper understanding of informed assent forms and to evaluate alignment with existing guidelines.

**Material and methods:**

The KIDS Madrid Young People Advisory Group (YPAG) reviewed six pediatric oncology trial assent forms using a structured questionnaire. Investigators provided item-specific weightings. Quantitative and qualitative data were integrated.

**Results:**

The analysis showed moderate satisfaction overall. Strengths included clarity in describing the trial purpose and risks/benefits. Key deficiencies were found in accessibility features such as audiovisual aids, and simplified language. Investigators prioritized technical accuracy, while KIDS emphasized clarity, structure, and engagement. Gaps were identified in adherence to existing guidelines, especially regarding document length, readability, and support resources.

**Discussion:**

There is a clear mismatch between investigators’ focus and young patients’ needs. While technical content was generally adequate, critical elements for child comprehension were lacking. These findings support the need to involve YPAGs to improve pediatric assent documents.

## Introduction

Pediatric drug research is the scientific, ethic, and legal method for evaluating the safety and efficacy of medications in infants and children. The process of obtaining consent and assent for pediatric clinical trials requires careful consideration, as both the parents and the child must understand the information and agree to participate (Hester and Miner, 2024; Tait and Geisser, 2017). Providing information to children must be tailored to their age and developmental stage, ensuring that they can comprehend the details, and that the informational materials are specifically designed for that (Waligora et al., 2014; Cayouette et al., 2022).

In 2020, 11% of clinical trials globally involved pediatric populations, emphasizing the need for robust patient information sheets that balance adolescents’ active participation with the necessary parental consent (Lepola et al., 2022; Grady et al., 2014; European Union, 2007). Understanding the factors that motivate or discourage children and their parents from participating in clinical trials is critical to the assent and consent process (Tromp et al., 2016). However, the complexity of assent and consent forms often hinders meaningful comprehension, particularly for children. Studies have shown that while simplification efforts may reduce complexity, overly intricate forms still fail to enhance understanding (Abdel-Rahman, 2019). Furthermore, these materials must reflect children’s perspectives to ensure their unique needs and views are meaning (Madden et al., 2016).

While the literature has extensively addressed informed consent, much of this work focuses on adult populations (Ruiz Escrivá, 2021; Giménez et al., 2016; Marrero-Álvarez et al., 2013; Karbwang et al., 2018) and there is a noticeable gap in research specifically reviewing the content and design of assent documents aimed at children.

Directly involving pediatric patients in the design of patient documents ensures their understanding of the information provided and enhances their ability to make autonomous decisions (Plataforma Tecnológica Española de Medicamentos Innovadores, 2021). This practice addresses ethical concerns surrounding parental consent and child assent, promoting the child’s right to participate meaningfully in decisions that affect them (Spriggs, 2023; Cotrim et al., 2021; O’Lonergan and Forster-Harwood, 2011). Such involvement also prevents the “diffusion of responsibility” between parents and adolescents, which can otherwise undermine the decision-making process (Annett et al., 2017)

Young People Advisory Groups (YPAGs) are invaluable forums where young individuals contribute their perspectives to enhance the relevance, quality, and inclusiveness of clinical research. Despite their potential, YPAGs remain underutilized. A recent study found that less than 1% of empirical studies included advice from YPAGs, underscoring their limited integration into clinical research design and implementation (Sellars et al., 2021).

Our study focuses on the evaluation of clinical trial assent documents conducted by a YPAGs setting in Madrid, Spain, at one of Europe’s leading Early Phase Clinical Trial Units for children with cancer. Our primary goal is to identify potential barriers to children’s proper understanding of the informed assent forms used in early-phase cancer clinical trials, and to evaluate how well current practices align with existing guidelines, including those developed by other YPAG groups and incorporated into the standards of the Spanish Agency of Medicines and Health Products (AEMPS) (Kids Barcelona, 2017; AEMPS, 2025).

## Material and methods

### Design

This is an explanatory sequential mixed-methods study that integrates quantitative and qualitative data. Quantitative phase was conducted first, followed by a qualitative phase to help interpret and explain the numerical results.

We used an explanatory sequential mixed-methods design to first create a standardized, cross-document profile of suitability/compliance and then use youth narratives to explain patterns and shape concrete recommendations. We did not choose a concurrent or qualitative-first approach because we needed upfront quantitative results to target qualitative probes to low-scoring domains and to ensure an *a priori*, comparable metric across documents.

### Participant

The KIDS Madrid group was established in 2018 at the National Children’s Hospital Niño Jesús, in accordance with the standards of the International Children’s Advisory Network (ICAN) (iCAN Research, 2014). After pandemic new participants selection had to be done, recruitment followed ICAN guidelines and was coordinated by the KIDS Madrid lead (AM). Following selection, all members completed a 1-year training program on clinical research fundamentals to ensure meaningful and informed participation. It comprises 14 adolescents aged 14–18 (57% male, 43% female), of whom 64% had a medical diagnosis and 36% were healthy volunteers–reflecting the diversity recommended for Young Persons Advisory Groups (YPAGs) (iCAN Research, 2014) The inclusion of healthy participants provides a broader perspective on pediatric healthcare, complementing the experiences of chronically ill youth. Among the group, four participants had a history of pediatric cancer, and four had taken part in clinical trials. These backgrounds can shape how assent materials are appraised, those familiar with trials may focus more on procedural details, whereas trial-naïve or healthy participants may rely more on plain language, and visual supports. This diversity was sought to capture potentially different perspectives that inform document design and to mirror real-world audiences, since in practice these materials are presented to adolescents with chronic illnesses or prior trial exposure as well as to those without such experiences.

All members were fluent in Spanish, and all assent forms reviewed during the study were written in Spanish

### Questionnaire used

A custom questionnaire developed specifically for this study was used (Supplementary Annex I). The design was based on the Suitability Assessment of Materials (SAM) by Doak et al. (1996) combined with elements from the study on consent evaluation by Matsui et al. (2012). The questionnaire comprised 30 closed-ended questions with the possibility of adding free text comments and was divided into two sections in line with these two main objectives of our study. Block A explores potential comprehension barriers, whereas Block B acts as a compliance audit of previously published KIDS/AEMPS recommendations, verifying their current adoption rather than their perceived usefulness. Block A contained 15 questions addressing the content required by European regulatory guidelines (Lepola et al., 2022; European Union, 2007) for pediatric clinical trial assents. Block B included 15 questions stemming from the recommendations published by the KIDS Barcelona group in 2017, subsequently integrated into the AEMPS guidelines (Kids Barcelona, 2017; AEMPS, 2025) Each question was evaluated on a three-point scale to simplify scoring and enhance comprehension among adolescents. Zero indicated that the content was not acceptable, 1 indicated that it could be improved, and 2 indicated perfect compliance. Additionally, questionnaire items are grouped into four latent variables (Supplementary Annex II): Clarity of trial information, comprehensibility of patient impact, accessibility of the document, and presence of additional resources.

### Assents included in the analysis

The review included the 6 informed assent documents for patients aged 12–17 years, from the 6 most recruited pediatric cancer phase I-II clinical trials during 2023, 4 industry sponsored and 2 academic sponsored. To ensure reflection of the contemporary practices, the most recent versions were the ones included in the analysis, and all had been created after 2021. To ensure confidentiality, identifying information such as sponsor details and clinical trial identifications were removed from the documents.

All assent documents were provided in Spanish, the mother tongue of all of the reviewers. The number of pages of the assents varies from 3 to 13, with an average of 8.6 pages.

### Document review process

Each participant independently reviewed the assent information sheets during three structured meetings facilitated by the KIDS Madrid coordinator that held between March and May 2024. Each session lasted 2 h, divided into 50-minute reviews of two assent documents, with a 20-min rest interval.

Prior to the meetings, participants received an introduction explaining the purpose of the study and detailed instructions on conducting the review. During the sessions, participants were encouraged to ask procedural questions but were not provided with specific content details about the documents.

Each KIDS member completed a total of 6 questionnaires, one for each of the assents they reviewed. This means that for each of the 30 questions in the questionnaire, each participant provided 6 responses, resulting in a total of 84 evaluations per question.

### Weighting of variables

The weighting process involved the three principal investigators from the clinical trials included in the study. Each investigator independently distributed a total of 90 points across the 30 questionnaire items according to perceived relevance. These weightings were then averaged to determine a consensus score per item.

### Quantitative statistical analysis

Ordinal variables were summarized using absolute, relative, and cumulative frequencies, while quantitative data were analyzed through means, quartiles, and standard deviations.

### Qualitative analysis

Free-text responses from participants were systematically analyzed using a content analysis approach. All qualitative data were first transcribed and imported into Microsoft Excel, and then independently reviewed by two analysts with experience in pediatric research and qualitative methods. The analysts conducted open coding, identifying recurring ideas and assigning preliminary labels to meaningful segments of text.

A coding framework was then iteratively developed based on emergent patterns. Although no pre-established coding tree was used, the categories were refined through successive rounds of discussion and comparison between analysts. Inter-coder agreement was assessed manually through side-by-side comparison of independently coded segments, and discrepancies were resolved through consensus in regular meetings.

Key themes were identified once no new codes emerged, indicating thematic saturation had been reached. Given the limited but focused nature of the qualitative data (i.e., short comments linked to structured questionnaire items), saturation was assessed not by the total volume of responses but by repetition and redundancy of themes across multiple items and participants. Representative verbatim quotes were selected to illustrate major themes, enhance validity, and provide context for the quantitative findings.

## Results

### Quantitative data analysis

The quantitative analysis revealed an overall mean score of 1.15 across all items, reflecting a moderate level of satisfaction with the content and design of the pediatric assent documents. Higher scores corresponded to items that largely met the expectations of KIDS evaluators, whereas lower scores highlighted significant deficiencies (Table 1)

**TABLE 1:** Ranking of items by increasing need for improvement based on KIDS Madrid scores (n = 84 evaluations per item).

Item	N	Mean	Median	SD	IQR	Score order subjects
q1A Is it clear why the clinical trial is being done and what disease it is aimed at?	84	1.83	2	0,406	0	1
q3A Is it clear what tests will be performed on patients within the trial?	84	1.79	2	0,441	0	2
q7A Is it clear that the child/young person does not have to participate if he/she does not want to and that there is nothing wrong if he/she chooses not to?	84	1,77	2	0,475	0	3
q5A Are the risks and benefits that may occur to patients from participating in the study clear?	84	1.76	2	0,456	0	4
q11B Do they address the child/young person in the second person singular?	84	1.75	2	0,557	0	5
q8B Does it include clear information on the hospital where it is carried out and contact details of the principal investigator?	84	1.71	2	0,572	0	6,5
q9B Is the term “patient”, or “child/youth” used instead of the term subject?	84	1.71	2	0,572	0	6,5
q7B Does it explain the possible side effects of the drug in a simple way?	84	1.63	2	0,555	1	8
q11A Does it state clearly that sometimes the patient may gain nothing by participating in the trial?	84	1.61	2	0,621	1	9
q12A Is it clear what will be done with the biological samples (blood, tissues, etc.) taken during the trial?	84	1.51	2	0,768	1	10
q4B Is the font and font size appropriate?	84	1.5	2	0,685	1	11
q6A Is it clear which drug is to be administered, and if other studies have been done with it before?	84	1.49	2	0,649	1	12
q14A Did you get all the information you needed to make a good decision about participating in the clinical trial??	84	1.46	2	0,63	1	13
q1B Is the language easy to understand?	84	1.39	2	0,695	1	14
q15A Do you consider that in general the document is written in a way that a child/young person of your age can easily understand?	84	1.35	2	0,752	1	15
q10A Is it clear when the trial will end?	84	1.25	1	0,79	1	16
q9A Do you think a child/youth would understand how long the study lasts, how many times he/she will have to go to the center and how long he/she will be there for each visit?	84	1.21	1	0,713	1	17,5
q2B Does this document have only the information we need and is it explained in a short, easy-to-understand way?	84	1.21	1	0,746	1	17,5
q2A Is it clear why it is necessary to do the clinical trial in children/adolescents?	84	1.15	1	0,752	1	19
q10B Does it include information on what to do in case of emergency, who to notify, how to proceed?	84	1.12	1	0,842	2	20
q3B Is it of reasonable length, 2-5 pages?	84	1.06	1	0,827	2	21
q8A Is there sufficient information explaining what other treatments could be followed if the patient decides not to participate in the study?	83	1.05	1	0,795	2	22
q5B Explain in general terms what a clinical trial is?	84	1.01	1	0,857	2	23
q4A Is it clear how participating in the study will affect the patient’s daily life: time to be able to go to school and to be with friends?	84	0.99	1	0,752	2	24
q14B Does it include a schedule of clinical trial activities?	84	0.8	0	0,889	2	25
q13A Is the role of the legal representative clear and what should be done when the patient reaches the age of majority, i.e., when he/she turns 18 years old?	83	0.72	0	0,816	1	26
q12B Does it use color, drawings, photos or diagrams to help understand the information?	84	0.51	0	0,703	1	27
q15B Does it include a free space for the child/young person to take notes?	84	0.48	0	0,784	1	28
q6B Does it include a list (glossary) with definitions of terms that are difficult to understand outside the healthcare field?	84	0.26	0	0,583	0	29
q13B Does it include additional audio-visual resources such as a video, a cartoon?	84	0.01	0	0,109	0	30

N, number of responses; SD, standard deviation; IQR, interquartile range. Items were scored on a 0–2 scale by the Young Persons’ Advisory Group (KIDS Madrid): 0 = unacceptable, 1 = needs improvement, 2 = optimal. Columns show N (number of evaluations), Mean, Median (P50), SD (standard deviation), and IQR (interquartile range, P75–P25). Lower rank numbers indicate less need for improvement; ties share the same rank.

Items with a mean score above 1.5, were deemed satisfactory and required minimal improvement. For instance, the clarity of the trial’s purpose (q1A) received a score of 1.8, indicating that the evaluators found this aspect clearly articulated. Similarly, the description of risks and benefits (q7A) scored 1.7, reflecting adequate information in these sections.

Items with mean scores ranging from 1.0 to 1.5, were considered moderately satisfactory but showed potential for enhancement. Notable examples included the adequacy of information needed to make an informed decision (q14A), which scored 1.4 but could be more engaging, and the readability and language appropriateness (q1B and q15A), which scored 1.3, suggesting opportunities for simplification or better adaptation to younger audiences.

Items with mean scores below 1.0, exposed critical deficiencies requiring substantial revisions. For example, glossary inclusion (q6B) scored only 0.2, emphasizing the necessity of a terminology guide to aid comprehension, while audiovisual resources (q13B) scored 0.01, highlighting the absence of visual aids to simplify complex concepts.

Supplementary Annex III includes and independent analysis done by the KIDS Madrid group coordinator about those items with more improvement needs and the presence at a document level within the six anonymized informed assent evaluated.

### Investigator weighting and priority analysis

No significant inter-investigator discrepancies were noted, and the process was consistent across all six assent documents evaluated. The principal investigators priority ranking placed the greatest emphasis on items related to the trial’s purpose, risks, and benefits (q1A, q5A, q7A). In contrast, accessibility features such as visual aids (q13B), glossary definitions (q6B), and formatting elements (q8B) received less attention, as they were deemed peripheral to the primary purpose of assent documents (Table 2).

**TABLE 2:** Subjective weighting of item importance by the three principal investigators.

Item	Weighting 1	Weighting 2	Weighting 3	Mean	SD	Weighting investigators	Weighting order
q15A Do you consider that in general the document is written in a way that a child/young person of your age can easily understand?	5	4	6	5,00	1	1,667	1,5
q7B Does it explain the possible side effects of the drug in a simple way?	5	4	6	5,00	1	1,667	1,5
q1A Is it clear why the clinical trial is being done and what disease it is aimed at?	6	4	2	4,00	2	1,333	5,5
q5A Are the risks and benefits that may occur to patients from participating in the study clear?	6	3	3	4,00	1.73	1,333	5,5
q7A Is it clear that the child/young person does not have to participate if he/she does not want to and that there is nothing wrong if he/she chooses not to?	4	4	4	4,00	0	1,333	5,5
q11A Does it state clearly that sometimes the patient may gain nothing by participating in the trial?	3	4	5	4,00	1	1,333	5,5
q14A Did you get all the information you needed to make a good decision about participating in the clinical trial?	6	4	2	4,00	2	1,333	5,5
q2B Does this document have only the information we need and is it explained in a short, easy-to-understand way?	4	4	4	4,00	0	1,333	5,5
q3A Is it clear what tests will be performed on patients within the trial?	2	4	5	3,67	1.53	1,222	10,0
q4A Is it clear how participating in the study will affect the patient’s daily life: time to be able to go to school and to be with friends?	5	3	3	3,67	1.15	1,222	10,0
q6A Is it clear which drug is to be administered, and if other studies have been done with it before?	3	4	4	3,67	0.58	1,222	10,0
q1B Is the language easy to understand?	3	4	3	3,33	0.58	1,111	12,5
q10B Does it include information on what to do in case of emergency, who to notify, how to proceed?	3	3	4	3,33	0.58	1,111	12,5
q9A Do you think a child/youth would understand how long the study lasts, how many times he/she will have to go to the center and how long he/she will be there for each visit?	2	4	3	3,00	1	1,000	15,0
q3B Is it of reasonable length, 2-5 pages?	3	3	3	3,00	0	1,000	15,0
q12B Does it use color, drawings, photos or diagrams to help understand the information?	5	2	2	3,00	1.73	1,000	15,0
q2A Is it clear why it is necessary to do de clinical trial in children/adolescents?	3	3	2	2,67	0.58	0,889	18,0
q5B Explain in general terms what a clinical trial is?	4	3	1	2,67	1.53	0,889	18,0
q14B Does it include a schedule of clinical trial activities?	2	1	5	2,67	2.08	0,889	18,0
q8A Is there sufficient information explaining what other treatments could be followed if the patient decides not to participate in the study?	1	3	3	2,33	1.15	0,778	22,5
q10A Is it clear when the trial will end?	1	4	2	2,33	1.53	0,778	22,5
q12A Is it clear what will be done with the biological samples (blood, tissues, etc.) taken during the trial?	1	3	3	2,33	1.15	0,778	22,5
q13A Is the role of the legal representative clear and what should be done when the patient reaches the age of majority, i.e., when he/she turns 18 years old?	2	2	3	2,33	0.58	0,778	22,5
q6B Does it include a list (glossary) with definitions of terms that are difficult to understand outside the healthcare field?	3	1	3	2,33	1.15	0,778	22,5
q11B Do they address the child/young person in the second person singular?	2	3	2	2,33	0.58	0,778	22,5
q8B Does it include clear information on the hospital where it is carried out and contact details of the principal investigator?	1	3	2	2,00	1	0,667	26,5
q9B Is the term “patient”, or “child/youth” used instead of the term subject?	1	3	2	2,00	1	0,667	26,5
q4B Is the font and font size appropriate?	2	2	1	1,67	0.58	0,556	28,0
q13B Does it include additional audio-visual resources such as a video, a cartoon?	1	1	1	1,00	0	0,333	29,0
q15B Does it include a free space for the child/young person to take notes?	1	0	1	0,67	0.58	0,222	30,0
Total	90	90	90	90		30,000	

SD, Standard deviation. Each investigator distributed 90 points across the 30 items; the table displays per-investigator weights, the Mean and SD across investigators, and the resulting Weighting order (lower numbers = higher importance; ties share rank).

### Latent variables

The clarity of clinical trial information, received positive evaluations from KIDS Madrid and was considered highly important by investigators, indicating minimal improvement needs. The second variable, comprehensibility of patient impact, was deemed highly important by investigators but moderately aligned with KIDS evaluations, highlighting areas such as detailed explanations of how the trial affects daily life requiring more attention. The third variable, document accessibility, was rated as a minor concern by investigators but highlighted by KIDS as needing moderate improvements, particularly in textual and visual clarity. Lastly, the presence of additional resources was identified as a critical deficiency by KIDS due to the absence of glossaries, audiovisual aids, and spaces for participant questions. This contrasted starkly with the minimal importance assigned to this category by investigators, underscoring the misalignment in priorities.

Table 3 and Supplementary Annex IV provide a detailed comparison of these latent variables.

**TABLE 3:** Comparison between KIDS’ perceived need for improvement and investigators’ assigned importance, grouped by latent variables.

Latent variables	Average order score KIDS	Average order valuation investigators
1 “Clarity of Clinical Trial Information”	12,61 (lower improvement need)	14,11 (moderate importance)
3 “Document accessibility”	12,86 (intermediate improvement need)	15,93 (minor importance)
2 “Understanding the impact on the patient”	13,71 (intermediate improvement need)	10,43 (high importance)
4 “Additional resources presence”	23,64 (High improvmente need)	21,93 (low importance)

The table compares the average ranking positions of item groups as perceived by the KIDS (based on need for improvement) and by the researchers (based on importance). Higher average rankings from the KIDS indicate that the group of items is perceived as more in need of improvement. Conversely, higher rankings from the researchers reflect lower importance assigned to those items. A visible inverse relationship suggests that items considered less important by researchers are often seen by the KIDS as needing greater improvement.

### Qualitative data analysis

Qualitative responses indicated that 85% of participants found the purpose of the trial and the targeted disease clearly explained, while only 40% felt the necessity of including children/adolescents was adequately justified. Similarly, while 90% considered the description of medical tests comprehensive, only 35% believed the impact on daily life (e.g., school attendance, social activities) was sufficiently addressed. One participant noted, “It tells me what might happen to me, but not how it will affect my daily life,” illustrating the central concern that assent documents often fail to address how participation could disrupt school or social routines. Another stated, “I understand the procedures, but I don’t know if I will be able to continue my normal activities,” reinforcing the theme that the practical implications of trial participation on quality of life are insufficiently detailed.

The analysis also highlighted that 70% of respondents understood the risks and benefits, but only 45% found information on alternative treatments clearly detailed. Additionally, 60% of participants struggled with the extensive paragraphs and lack of visual aids, suggesting a need for more structured and engaging formats such as tables or infographics. One participant suggested, “It would be easier to understand if there were a calendar or table showing how many visits I would have and when,” directly supporting the recommendation to use visual formats for clarity. Another commented, “There is too much text in some parts; it would help if the information was broken into sections or had more images,” which underscores the need for better organization and visual enhancement to improve accessibility. Furthermore, while 75% found the language generally accessible, some reported difficulties with technical terminology, emphasizing the need for a glossary. A respondent commented, “There are some complicated words that I don’t understand, and I’m not sure where to look them up,” highlighting the problem of jargon that limits comprehension. Another added, “Some medical terms are confusing. Maybe a short explanation or a list of definitions would help,” aligning with the recommendation to include a glossary or explanatory notes to support understanding.

These findings highlight recurring shortcomings in clarity, format, and accessibility of assent documents, which directly affect adolescents’ ability to understand and evaluate trial participation.

## Discussion

To our knowledge, this is the first report from a group of young advisory KIDS reviewing informed assents for early-phase clinical trials in pediatric cancer and providing their perspective. This study highlights the persistent gap between investigators’ and children’s priorities, underscoring the need to include young advisory groups in the design and development of pediatric patient information forms. While investigators focused on technical clarity and compliance with regulatory guidelines, the KIDS advisory group highlighted the necessity for additional tools, such as glossaries, diagrams, and audiovisual aids, to enhance comprehension and engagement. In our evaluation by the KIDS Madrid group, the mandatory content of pediatric assent documents generally received favorable assessments. These documents included sufficient information about trial rationale, testing procedures, risks, benefits, and voluntary participation, and they clearly articulated the potential lack of personal benefit for participants.

However, certain deficiencies were noted, such as insufficient details on alternative treatment options, the rationale for pediatric involvement, comprehensive explanations of the investigational drug, trial duration, end-of-trial procedures, and overall writing style. Young evaluators emphasized the importance of ensuring age-appropriate language and accessibility, highlighting the need for clearer information about the trial’s impact on daily life and the role of legal representatives.

Meanwhile, compliance with the items derived from the 2017 KIDS Barcelona (Kids Barcelona, 2017) recommendations revealed moderate to critical improvement needs, particularly regarding document length, font size, language suitability, emergency instructions, and the inclusion of glossaries, audiovisual resources, and diagrams.

The absence of visual aids and simplified language in current assent documents in our study resonates with Villamañán et al., 2016, who noted that overly complex and lengthy documents hinder understanding and undermine the ethical principles of informed consent. Our findings align also with previous reports, that stressed the critical role of youth advisory groups in co-designing materials and adapting language and format to enhance relevance and accessibility (Cayouette et al., 2022; Ruiz Escrivá, 2021; Sellars et al., 2021).

The notably low scores for audiovisual resources in our evaluation reinforce the need to incorporate such tools, that enhance both understanding and engagement in pediatric research (O’Lonergan and Forster-Harwood, 2011; Antal et al., 2017). This is further supported by evidence on the benefits of using illustrations, narrative approaches, and plain language to improve comprehension (Abdel-Rahman, 2019; Soll et al., 2020).

The qualitative phase plays a crucial role in contextualizing and interpreting quantitative findings. Although the number of qualitative responses is limited, it adds substantial value by highlighting individual perspectives and enriching the overall analysis. These findings suggest that, although the document effectively conveys essential aspects of the trial, improvements in visual presentation, structural organization, and additional details on daily life impact and alternative treatments would enhance its comprehensibility and usability for young participants.

To our knowledge, there are no published evaluations from other KIDS YPAGs specifically assessing pediatric assent forms, limiting direct cross-country benchmarking. Nonetheless, the needs identified here, cohere with health-literacy and youth-involvement principles cited.

To translate these findings into practice, Table 4 summarizes concrete changes directly derived from our results, each mapped to the specific gap observed.

**TABLE 4:** Practical recommendations mapped to study findings.

Gap observed	Evidence	Practical recommendation	Example artifact
Daily-life impact insufficient	Only 35% of participants felt the impact on school, social life, and daily routines was clearly addressed; quotes explicitly asked for more concrete day-to-day information.	Add a dedicated “What will my days look like?” section linking visits and procedures to daily activities.	1-page schedule with notes on school/social adjustments.
Alternatives not clearly presented	Only 45% considered information on alternative treatments clear; participants reported uncertainty about options beyond trial participation.	Include a plain-language comparison of options.	3–5 row table comparing trial, standard care, and other options.
Glossary missing	The glossary received one of the lowest mean scores (0.2/2); several participants reported difficulty understanding technical terms and asked for definitions.	Provide a child-friendly glossary and link key terms inline.	A–Z list with ≤20-word definitions; inline tooltips.
Visual aids lacking	Audiovisual resources were virtually absent (mean 0.01/2); 60% struggled with long text and the lack of visuals; participants suggested calendars/tables and simple images.	Add visuals for the most complex concepts and timelines.	Procedure flow diagram; icon-based risk/benefit summary; short explainer video.
Readability/length issues	Readability and language appropriateness were only moderate (means around 1.3/2); 60% noted overly long paragraphs and dense text.	Chunk content with headings and bullets; set plain-language targets.	Micro-style guide (max sentence length; reading-level goal).
Visit schedule hard to visualize	While 90% found test descriptions comprehensive, participants still asked for a clearer overview of “how many visits and when.”	Summarize visits, tests, and timing in one place.	Calendar or Gantt-style timeline of visits.
Roles and decision-making unclear	Youth feedback indicated confusion about the roles of children vs. parents/legal representatives and where to ask questions.	Clarify roles and rights in a concise box.	“Who does what?” call-out with contact points.
Trial duration and end-of-trial procedures under-specified	Document review identified insufficient detail on overall duration and what happens after the trial ends.	Add a “What happens next?” section.	Checklist for end-of-trial steps and follow-up.
Formatting/layout not salient	Youth highlighted layout clarity as a need; readability remained only moderate.	Set minimum layout standards to aid scanning.	One-page visual template (font size, spacing, call-outs).

Despite recent advancements, challenges persist: information on treatment options and trial duration is often lacking, emphasizing the need for more standardized explanations in pediatric research (Lombardi et al., 2018). Shorter, focused documents can improve comprehension without sacrificing essential content (Matsui et al., 2012), and incorporating real-time discussions, along with opportunities for children to ask questions, further enhances understanding while reducing anxiety (Annett et al., 2017).

These improvement needs are aligned with the principle that inadequate assent documents present substantial ethical and regulatory concerns, particularly regarding pediatric autonomy. Although minors cannot provide legal informed consent, ethical guidelines mandate their involvement in research decisions proportionate to their maturity. This principle is consistent across several international and national frameworks.

The WMA Declaration of Helsinki (World Medical Association, 2013) underscores the ethical imperative to seek assent from participants incapable of providing informed consent, alongside consent from their legal guardians. This obligates researchers to genuinely inform children and respect their evolving autonomy, moving beyond mere symbolic gestures.

Similarly, the European Union (2014) mandates that information provided to minors be “adapted to their age and mental maturity.” Thus, deficient assent materials–lacking clarity on investigational treatments, trial procedures, post-trial care, or using inaccessible language–are not only ethically problematic but also risk non-compliance with EU regulations.

In the U.S. Government Publishing Office (2025), 45 CFR 46, Subpart D, which governs research involving children, requires adequate provisions for soliciting assent when deemed appropriate by the Institutional Review Board (IRB). Guidance from the Office for Human Research Protections (OHRP) emphasizes that assent extends beyond a signature, necessitating affirmative agreement based on understandable information. Consequently, overly complex, lengthy, or irrelevant assent materials risk reducing assent to a token gesture, failing to meet regulatory intent.

To be sure patients are able to make well informed decisions, it is necessary to use materials appropriate to their level of comprehension, in order to preserve their autonomy during the process (Lombardi et al., 2018; Scherer et al., 2013)

This study has several noteworthy limitations. First, all assessments were conducted in a single pediatric oncology center, which narrows the applicability of our findings to other settings. Second, the evaluations performed by the KIDS Madrid group are inherently subjective and may reflect the specific backgrounds and experiences of its members.

Third, the questionnaire utilized in this investigation, while derived from the well-established Suitability Assessment of Materials (SAM) Doak et al. (1996) and the framework developed by Matsui et al. (2012) did not undergo a formal psychometric validation process. The adaptation aimed to create a context-specific instrument tailored for an adolescent population, given that the original frameworks were primarily designed for adult audiences in clinical or educational settings. Consequently, modifications to item wording and format were implemented to enhance age-appropriate comprehension and relevance.

To further facilitate accessibility and reduce cognitive load for younger respondents, a simplified three-point Likert scale (0 = unacceptable, 1 = needs improvement, 2 = optimal) was employed. Adolescents may encounter difficulties with more nuanced Likert formats, particularly concerning subtle or abstract distinctions between closely spaced response options. The simplified scale was intended to promote more reliable self-reporting.

However, this methodological adaptation introduces several limitations. Foremost, the reduced number of response options inherently constrains the granularity of the data, potentially obscuring subtle distinctions in participant evaluations. Furthermore, the inclusion of a middle option (“needs improvement”) may have induced a central tendency bias, whereby respondents might have defaulted to this neutral choice when uncertain. This potential bias could lead to an underestimation of both highly positive and highly negative perceptions.

Given the absence of a formal psychometric validation process for the adapted questionnaire–specifically, assessments of construct validity, internal consistency, or test-retest reliability–caution is warranted when interpreting the results derived from this instrument. Although the content was based on validated frameworks and reviewed by subject-matter experts to establish face and content validity, future research endeavors should prioritize rigorous validation procedures to ascertain the psychometric properties of this adapted instrument. In addition, it has been attempted to address this through the collection of qualitative data, and the research was continued with a focus group (study in press) to triangulate the results. In addition, the small sample size and the static nature of the documents reviewed limit our ability to capture the full spectrum of current practices in assent-form development. Finally, our cross-sectional evaluation included only adolescents and did not capture parents’ perspectives, which are central to the assent process; therefore, the findings may not generalize to younger children or reflect family decision dynamics. Future studies should include parents/guardians and younger age bands using age-tailored formats, and, where feasible, assess post-exposure outcomes longitudinally to determine the impact of revised materials.

In conclusion, our study reinforces the necessity of a paradigm shift in designing pediatric assent documents. Our findings, consistent with existing literature, highlight the critical need for patient-centered approaches, such as the integration of multimedia tools and the active involvement of young advisory groups like KIDS. The integration of a qualitative phase in our study ensures a more complete and meaningful interpretation of the data, which reinforces the conclusions of the study. By addressing both the technical and experiential needs of pediatric participants, we can create assent documents that are not only compliant with regulatory standards but also accessible, engaging, and effective in supporting informed decision-making.

## Data Availability

The raw data supporting the conclusions of this article will be made available by the authors, without undue reservation.
