# Supplementary material for: Analysis of pediatric assent information in early-phase cancer clinical trials through a children’s research advisory group
Source: Front Psychol. 2025 Oct 3;16:1655835. doi: 10.3389/fpsyg.2025.1655835 (PMC12533476; doi:10.3389/fpsyg.2025.1655835)
Supplement: Supplementary file 2 [file Table_2.docx]

**Annex II:** Item-to-latent-variable mapping (30 items across four constructs). Lists the questionnaire items grouped into: (1) Clarity of clinical trial information, (2) Understanding the impact on the patient, (3) Document accessibility, and (4) Presence of additional resources.

***1. Clarity of clinical trial information”***

- *1A. Is it clear why the clinical trial is being conducted and for which disease?*
- *2A. Is it clear why it is necessary to conduct the trial in children/adolescents?*
- *3A. Is it clear what tests will be performed on the patients within the trial?*
- *6A. Is it clear which drug is to be administered, and whether other studies have been done with it before?*
- *9A. Do you think a child/young person would understand how long the study lasts, how many times he/she will have to go to the center, and how long he/she will be there for each visit?*
- *10A. Is it clear when the trial will end?*
- *12A. Is it clear what will be done with the biological samples (blood, tissue, etc.) that will be taken during the trial?*
- *14 A. Did you get all the information you needed to make a good decision about participating in the clinical trial?*
- *5B. Explain in general terms what a clinical trial is?*

***2. Understanding the impact on the patient”***

- *4A. Is it clear how participating in the study will affect the patient's daily life: time to go to school, time to spend with friends?*
- *5A. Is it clear what risks and benefits may occur to the patient by participating in the study?*
- *7A. Is it clear that the child/young person does not have to participate if he/she does not want to and that there is nothing wrong if he/she decides not to?*
- *8A. Is there sufficient information explaining what other treatments might be followed if the patient decides not to participate in the study?*
- *11A. Does it clearly state that sometimes the patient may not gain anything by participating in the trial?*
- *13A. Is the role of the legal representative clear and what should be done when the patient reaches the age of majority, i.e. when he/she turns 18 years of age?*
- *7B. Does it explain the possible side effects of the drug in a simple way?*

***3. Document accessibility***

- *15A. Do you think that overall the document is written in a way that a child/young person of your age can easily understand?*
- *1B. Is the language easy to understand?*
- *2B. Does this document have only the information we need and is it explained in a short, easy-to-understand way?*
- *3B. Is it a reasonable length, 2-5 pages?*
- *4B. Is the typeface and font size appropriate?*
- *9B. Is the term “patient”, or “child/youth” used instead of the term “subject”?*
- *11B. Is the child/young person addressed in second person singular?*

***4. Presence of additional resources***

## *8B. Does it include clear information on the hospital where it is being conducted and contact details of the principal investigator?*

## *6B. Does it include a list (glossary) with definitions of terms that are difficult to understand outside of the health care setting?*

## *10B. Does it include information on what to do in case of emergency, who to notify, how to proceed?*

## *12B. Does it use color, drawings, photos or diagrams to aid in understanding the information?*

## *13B. Does it include additional audiovisual resources such as a video, a cartoon?*

## *14B. Does it include a schedule of clinical trial activities?*

## *15B. Does it include a free space for the child/young person to take notes?*
