# Supplementary material for: Analysis of pediatric assent information in early-phase cancer clinical trials through a children’s research advisory group
Source: Front Psychol. 2025 Oct 3;16:1655835. doi: 10.3389/fpsyg.2025.1655835 (PMC12533476; doi:10.3389/fpsyg.2025.1655835)
Supplement: Supplementary file 3 [file Table_3.docx]

**ANNEX III**.: **Characteristics of the six anonymized informed assent documents reviewed (A–F, versions ≥2021).**

| **INFORMED ASSENT DOCUMENT** | **Document length in number of pages** | **Font size** | **Does it containts colors?** | **Does it contains visual aids to support written information** | **Includes clear Alternative treatments information** | **Impact on daily life explanations included** | **Glossaries presence** | **Rationale about the need to do the clinical trial in paediatric population** | **Clear information on study duration/end of study procedures included?** |
| --- | --- | --- | --- | --- | --- | --- | --- | --- | --- |
| **Informed Assent Document A** | 13 | Calibri 16 | Yes, but no colour printer at site. | Yes | No | No | No | No | No/No |
| **Informed Assent Document B** | 9 | Cambria 12 | No | No | No | No | No | Just because it only has been done in adults, nothing else mentioned. | Yes/No |
| **Informed Assent Document C** | 11 | Calibri 11 | No | No | Yes | No | No | Yes | No/No |
| **Informed Assent Document D** | 3 | ArialMT 11 | No | No | No | No | No | No | No/No |
| **Informed Assent Document E** | 11 | ArialMT 11 | A few, but no colour printer used | Just one image | Yes | No | No | No | Yes/No |
| **Informed Assent Document F** | 9 | ArialMT 11 | No | No | No | Yes | No | No | No |
