# Supplementary material for: Analysis of pediatric assent information in early-phase cancer clinical trials through a children’s research advisory group
Source: Front Psychol. 2025 Oct 3;16:1655835. doi: 10.3389/fpsyg.2025.1655835 (PMC12533476; doi:10.3389/fpsyg.2025.1655835)
Supplement: Supplementary file 4 [file Table_4.docx]

**ANNEX IV**.: **Item-level comparison of KIDS Madrid improvement needs vs investigators’ importance rankings.** The items are ranked by need for improvement from lowest to highest as per KIDS Madrid analysis. Results compared with the importance given to the items by the researchers.

| **ITEMS** | **Need for improvement according to KIDS evaluation** | **Investigators’ weighting.** |
| --- | --- | --- |
| **1A.** Is it clear why the clinical trial is being done and what disease it is aimed at? | Mean value. > 1.5 (NO NEED) | First 8 in importance (HIGH IMPORTANCE) |
| **3A.** Is it clear what tests will be performed on patients within the trial? | Mean value. > 1.5 (NO NEED) | Order of importance from 9 to 16 (MODERATE IMPORTANCE) |
| **5A.** Are the risks and benefits that may occur to patients from participating in the study clear? | Mean value. > 1.5 (NO NEED) | First 8 in importance (HIGH IMPORTANCE) |
| **7A.** Is it clear that the child/young person does not have to participate if he/she does not want to and that there is nothing wrong if he/she chooses not to? | Mean value. > 1.5 (NO NEED) | First 8 in importance (HIGH IMPORTANCE) |
| **11A.** Does it state clearly that sometimes the patient may gain nothing by participating in the trial? | Mean value. > 1.5 (NO NEED) | First 8 in importance (HIGH IMPORTANCE) |
| **12A.**  Is it clear what will be done with the biological samples (blood, tissues, etc.) taken during the trial? | Mean value. > 1.5 (NO NEED) | Order of importance from 17 to 25 (MINOR IMPORTANCE) |
| **7B.** Does it explain the possible side effects of the drug in a simple way? | Mean value. > 1.5 (NO NEED) | First 8 in importance (HIGH IMPORTANCE) |
| **8B.** Does it include clear information on the hospital where it is carried out and contact details of the principal investigator? | Mean value. > 1.5 (NO NEED) | Order of importance from 26 to 30 (LOW IMPORTANCE) |
| **9B.** Is the term “patient”, or “child/youth” used instead of the term subject? | Mean value. > 1.5 (NO NEED) | Order of importance from 26 to 30 (LOW IMPORTANCE) |
| **ITEMS** | **Need for improvement according to KIDS evaluation** | **Investigators’ weighting.** |
| **11B.**  Do they address the child/young person in the second person singular? | Mean value. > 1.5 (NO NEED) | Order of importance from 17 to 25 (MINOR IMPORTANCE) |
| **2A.** Is it clear why it is necessary to do de clinical trial in children/adolescents? | Mean > 1 y < 1,5 (MODERATE NEED) | Order of importance from 17 to 25 (MINOR IMPORTANCE) |
| **6A.** Is it clear which drug is to be administered, and if other studies have been done with it before? | Mean > 1 y < 1,5 (MODERATE NEED) | Order of importance from 9 to 16 (MODERATE IMPORTANCE) |
| **8A.** Is there sufficient information explaining what other treatments could be followed if the patient decides not to participate in the study? | Mean > 1 y < 1,5 (MODERATE NEED) | Order of importance from 17 to 25 (MINOR IMPORTANCE |
| **9A.** Do you think a child/youth would understand how long the study lasts, how many times he/she will have to go to the center and how long he/she will be there for each visit? | Mean > 1 y < 1,5 (MODERATE NEED) | Order of importance from 9 to 16 (MODERATE IMPORTANCE) |
| **10A.** Is it clear when the trial will end? | Mean > 1 y < 1,5 (MODERATE NEED) | Order of importance from 17 to 25 (MINOR IMPORTANCE) |
| **14A.** Did you get all the information you needed to make a good decision about participating in the clinical trial? | Mean > 1 y < 1,5 (MODERATE NEED) | First 8 in importance (HIGH IMPORTANCE) |
| **15A** Do you consider that in general the document is written in a way that a child/young person of your age can easily understand? | Mean > 1 y < 1,5 (MODERATE NEED) | First 8 in importance (HIGH IMPORTANCE) |
| **1B.** Is the language easy to understand? | Mean > 1 y < 1,5 (MODERATE NEED) | Order of importance from 9 to 16 (MODERATE IMPORTANCE) |
| **ITEMS** | **Need for improvement according to KIDS evaluation** | **Investigators’ weighting.** |
| **2B**  Does this document have only the information we need and is it explained in a short, easy-to-understand way? | Mean > 1 y < 1,5 (MODERATE NEED) | First 8 in importance (HIGH IMPORTANCE) |
| **3B** Is it of reasonable length, 2-5 pages? | Mean > 1 y < 1,5 (MODERATE NEED) | Order of importance from 9 to 16 (MODERATE IMPORTANCE) |
| **4B** Is the font and font size appropriate? | Mean > 1 y < 1,5 (MODERATE NEED) | Order of importance from 26 to 30 (LOW IMPORTANCE) |
| **5B**  Explain in general terms what a clinical trial is? | Mean > 1 y < 1,5 (MODERATE NEED) | Order of importance from 17 to 25 (MINOR IMPORTANCE) |
| **10B.** Does it include information on what to do in case of emergency, who to notify, how to proceed? | Mean > 1 y < 1,5 (MODERATE NEED) | Order of importance from 9 to 16 (MODERATE IMPORTANCE) |
| **4A.** Is it clear how participating in the study will affect the patient's daily life: time to be able to go to school and to be with friends?? | Mean > 0,5 y <1 (PRIORITY NEED) | Order of importance from 9 to 16 (MODERATE IMPORTANCE) |
| **13A.** Is the role of the legal representative clear and what should be done when the patient reaches the age of majority, i.e. when he/she turns 18 years old? | Mean > 0,5 y <1 (PRIORITY NEED) | Order of importance from 17 to 25 (MINOR IMPORTANCE) |
| **12B.**  Does it use colour, drawings, photos or diagrams to help understand the information? | Mean > 0,5 y <1 (PRIORITY NEED) | Order of importance from 9 to 16 (MODERATE IMPORTANCE)) |
| **14B.** Does it include a schedule of clinical trial activities? | Mean > 0,5 y <1 (PRIORITY NEED) | Order of importance from 17 to 25 (MINOR IMPORTANCE) |
| **6B** Does it include a list (glossary) with definitions of terms that are difficult to understand outside the healthcare field? | Mean < 0,5 (CRITICAL NEED) | Order of importance from 17 to 25 (MINOR IMPORTANCE) |
| **ITEMS** | **Need for improvement according to KIDS evaluation** | **Investigators’ weighting.** |
| **13B.** Does it include additional audio-visual resources such as a video, a cartoon? | Mean < 0,5 (CRITICAL NEED) | Order of importance from 26 to 30 (LOW IMPORTANCE) |
| **15B.** Does it include a free space for the child/young person to take notes? | Mean < 0,5 (CRITICAL NEED) | Order of importance from 26 to 30 (LOW IMPORTANCE) |
